# Effect of Yoga on Pain, Brain-Derived Neurotrophic Factor, and Serotonin in Premenopausal Women with Chronic Low Back Pain

**DOI:** 10.1155/2014/203173

**Published:** 2014-07-10

**Authors:** Moseon Lee, Woongjoon Moon, Jaehee Kim

**Affiliations:** ^1^Graduate School of Alternative Medicine, Kyonggi University, 24 Kyonggidae-ro 9-gil, Seodaemun-gu, Seoul 120-702, Republic of Korea; ^2^Division of General Studies, Seoil University, Yongmasan-ro 90-gil, Jungnang-gu, Seoul 131-702, Republic of Korea

## Abstract

*Background*. Serotonin and brain-derived neurotrophic factor (BDNF) are known to be modulators of nociception. However, pain-related connection between yoga and those neuromodulators has not been investigated. Therefore, we aimed to evaluate the effect of yoga on pain, BDNF, and serotonin. *Methods*. Premenopausal women with chronic low back pain practiced yoga three times a week for 12 weeks. At baseline and after 12 weeks, back pain intensity was measured using visual analogue scale (VAS), and serum BDNF and serotonin levels were evaluated. Additionally, back flexibility and level of depression were assessed. *Results*. After 12-week yoga, VAS decreased in the yoga group (*P* < 0.001), whereas it increased (*P* < 0.05) in the control group. Back flexibility was improved in the yoga group (*P* < 0.01). Serum BDNF increased in the yoga group (*P* < 0.01), whereas it tended to decrease in the control group (*P* = 0.05). Serum serotonin maintained in the yoga group, while it reduced (*P* < 0.01) in the control group. The depression level maintained in the yoga group, whereas it tended to increase in the control group (*P* = 0.07). *Conclusions*. We propose that BDNF may be one of the key factors mediating beneficial effects of yoga on chronic low back pain.

## 1. Introduction

The most recent systematic review of the global prevalence of low back pain in adults showed the 1-month prevalence of 23% [1], suggesting that low back pain is a global health problem. Indeed, it has been reported that prevalence of chronic low back pain ranges from 10 to 20% and recently increases [1, 2]. Approximately 90% of low back pain is nonspecific, defined as pain symptoms with no specific causes [3]. Overall, most of patients with back pain recover by 4–12 weeks and the pain tends to become chronic after 12 weeks [4, 5]. Chronic low back pain is recognized to have multifactorial etiology including mechanical, psychological, and neurological factors [6]. Therefore, multidisciplinary treatment approaches seem to be needed [6]. Nonpharmacological therapies such as exercise, acupuncture, massage, spinal manipulation, and yoga have been recommended and shown to be moderately effective for treating chronic low back pain [7–12].

Yoga, one of the most popular mind-body interventions, has been known to improve back pain and functional disability in patients with chronic low back pain [12, 13]. It is typically considered that yoga is comprised of physical postures (asanas), breathing techniques (pranayama), and meditation (dhyana) [14]. Recently, it is suggested that improvement of physical function and modulation in pain perception pathway may act synergistically [6, 15] although the underlying mechanisms of the effect of yoga on chronic low back pain still remain unclear [12].

Serotonin (5-hydroxytryptamine [5-HT]) has been relatively well known to be associated with nociception modulating the endogenous analgesic system [16]. Antidepressants have been treated for pain relief and reported to have antinociceptive effect partly mediated by the serotonergic systems [17, 18]. Recently, antidepressant treatment has been reported to reduce pain in nondepressed patients with nonneuropathic chronic low back pain [19].

Brain-derived neurotrophic factor (BDNF) which plays important roles in neuroplasticity of central nervous system is now known to regulate sensory neurotransmission at spinal and brain levels and to modulate neuropathic and inflammatory pain in adulthood [20–22]. Over the past decade, animal and human studies have been shown that BDNF is expressed in nondegenerate and degenerate intervertebral discs and herniated disc implicating that BDNF may be associated with low back pain [23–26].

BDNF and serotonin increase with exercise intervention and are regarded as mediating beneficial effects of exercise on mental and neurological disorders [20]. Although considerable evidences that exercise increases BDNF levels in various disease states have been accumulated [20, 27–29], the detection of changes in BDNF levels as an effector of exercise on pain modulation has not been reported [29]. Interestingly, one recent study reported that serum BDNF increased with 12-week yoga in depression patients [30]. Moreover, serotonin has been proposed as one of neuromodulators mediating the effect of yoga [31, 32]. Given previous findings, we hypothesized that yoga could change BDNF and serotonin levels and changes in those neuromodulators of pain could be concomitant with improvements in pain and physical function in patients with chronic low back pain.

To the best of our knowledge, the effect of yoga on any pain modulator including BDNF and serotonin in patients with low back pain has not yet been investigated. Accordingly, in this study we investigated effects of 12-week yoga intervention on pain, back flexibility, depression, and serum BDNF and serotonin levels in premenopausal women with chronic low back pain.

## 2. Methods

### 2.1. Study Design

Forty-three premenopausal women with chronic low back pain were recruited with a local flyer in the Dongtan city in Gyeonggi-do and Seoul city, Republic of Korea. Twenty-three women in an experimental group underwent a 12-week group-based yoga program. Twenty women in a control group who were matched to the experimental group by age and body mass index (BMI) were untreated. Age and BMI were matched in two groups since serum level of BDNF is age- and weight-dependent [33]. In addition, because benefits of exercise on BDNF were reported to depend on the presence of estrogen [34], we have excluded postmenopausal women from the study.

At baseline and after 12 weeks, back pain intensity, back flexibility, depression, and serum BDNF and serotonin levels were measured in both groups. Data from subjects who did not complete less than 70% of yoga sessions and dropped out due to personal reasons were excluded from data analysis. Finally, data from fourteen women in the yoga group and eleven women in the control group were analyzed. Attrition rate in the yoga and control groups was 61% and 55%, respectively.

Ethical approval for this study was obtained from Institutional Review Board of Korea National Institute for Bioethics Policy (IRB approval number PIRB12-069-02). All subjects gave their written informed consent before participating in study.

### 2.2. Subjects

The inclusion criteria were as follows: premenopausal women; low back pain lasting more than 3 months; nonobese (BMI < 25 kg/m^2^); no radiating pain; no spine surgery; no use of pain medication; and currently no regular exercise or other therapy for back pain. Exclusion criteria included pregnancy, inflammatory disease (e.g., rheumatoid arthritis), infectious disease, spine disorders, osteoporosis, cancer, cardiovascular disease, or neurological disease.

### 2.3. Yoga Program

The 12-week Hatha yoga program was performed for 1 hour, three times a week in a yoga studio. One experienced yoga instructor taught all yoga classes. The instructor has a long-term experience in teaching yoga therapy to individuals with low back pain. Yoga program consisted of a set of 10-minute warm-up (breathing and stretching), 40-minute yoga poses with appropriate awareness of breathing, and 10-minute relaxation and meditation. The Hatha yoga poses used in this study were adapted from previous studies [13, 35]. The yoga program was divided into three 4-week segments. Twelve poses were selected from 24 postures for each segment. Subjects were gradually progressed from simple poses to more challenging poses with each subsequent segment. No props were used. Some yoga poses were modified for people with low back pain. Participants were recommended to decrease the range of motion or fold their knees not to go over their physical limits. Plow pose and shoulder-stand pose were replaced with bridge pose when they had difficulties. Each segment included the following poses:

1. weeks 1–4: seated forward bend (Paschimottanasana), half-spinal twist pose (Ardha Matsyendrasana), cow face pose (Gomukhasana), cobra pose (Bhujangasana), heron pose (Krauncasana), fish pose (Matsyasana), tree pose (Vrksasana), cat and cow pose (Marjariasana), locust pose (Salabhasana), standing forward bend (Uttanasana), triangle pose (Utthita Trikonasana), and butterfly pose (Baddha Konasana);
2. weeks 5–8: tree pose (Vrksasana), cat and cow pose (Marjariasana), locust pose (Salabhasana), standing forward bend (Uttanasana), triangle pose (Utthita Trikonasana), butterfly pose (Baddha Konasana), half-moon pose (Ardha Chandrasana), bridge pose (Setu Bandhasana), bow pose (Dhanurasana), knee together twist (Jathara Parivartanasana), downward-facing dog (Adho Mukha Svanasana), and plow pose (Halasana);
3. weeks 9–12: half-moon pose (Ardha Chandrasana), bridge pose (Setu Bandhasana), bow pose (Dhanurasana), knee together twist (Jathara Parivartanasana), downward-facing dog (Adho Mukha Svanasana), plow pose (Halasana), gate pose (Parighasana), camel pose (Ustrasana), wide seated forward bend pose (Upavistha Konasana), warrior pose (Virabhadrasana), supine spinal twist (Supta Matsyendrasana), and shoulder-stand pose (Sarvangasana).

### 2.4. Baseline Characteristics

Body weight and height were measured, and BMI was calculated to estimate obesity. Previous treatments for low back pain, duration of back pain, diagnosed diseases from doctors, medications currently taking, and history of smoking were surveyed.

### 2.5. Outcome Measures

To evaluate the beneficial effect of our 12-week yoga program on back pain (as well as physical function and depression) at the molecular level, we measured the changes of BDNF and serotonin levels for the following reasons:

1. BDNF was implicated in playing a role in nociception of back pain [23–26];
2. BDNF was suggested to act cooperatively with serotonin [20];
3. Serotonin was proposed as a neuromodulator mediating the effect of yoga [31, 32];
4. Serotonin is involved both in pain and depression [16–19];
5. Both BDNF and serotonin increase with exercise intervention [20].

#### 2.5.1. Primary Outcome Variables

Primary outcomes were back pain intensity and serum BDNF and serotonin levels.

1. Back pain intensity was assessed with the visual analogue scale (VAS). Patients estimated their intensity of pain and placed a mark on a 10 cm horizontal line with descriptive phrases at either end such as “no pain” and “very severe pain” [36]. The distance from the “no pain” point was then measured (0–100 mm).
2. Blood was collected from the antecubital vein and then centrifuged. Serum was separated and stored in −80°C freezer. Serum BDNF and serotonin levels were analyzed by the Green Cross Reference Laboratory. Serum BDNF levels were measured with enzyme-linked immunosorbent assay (ELISA) using Human BDNF Immunoassay ELISA kits (R&D Systems, Minneapolis, MN, USA) and a microplate reader (VersaMax, Molecular devices, Sunnyvale, CA, USA) according to the manufacturer's instructions. By using high performance liquid chromatography (HPLC), serum serotonin levels were measured (Waters Alliance HPLC system, Waters Corporation, Milford, MA, USA). The levels of serum serotonin were calculated using a standard curve. Chromatographic separation was performed usingClinRep Serotonin column (Recipe, Munich, Germany) in combination with a Serotonin Kit (Recipe, Munich, Germany) according to the manufacturer's instructions.

#### 2.5.2. Secondary Outcome Variables

Secondary outcomes included back flexibility and severity of depression.

1. Back flexibility was assessed with the trunk flexion test. The trunk flexion test was performed to evaluate the flexibility of lower back and hamstring using a measurement box (OST-5103, Oseong, Republic of Korea). The subject stood with the knee straight on the box and bent toward the toes. The distances from the subject' fingertips to the floor were measured and the score for those who could not reach the toes was marked with minus sign.
2. Depressive symptoms were assessed by the Korean version of Beck Depression Inventory (BDI). The BDI evaluates 21 symptoms of depression and each symptom is rated on a 4-point intensity scale (0–3) and scores are added to give a total ranging from 0 to 63 with higher scores indicating greater depression. Scores 0–9 indicate minimal depression and 10–18 represent mild depression [37, 38]. The Korean version of the BDI has been validated previously [39, 40].

### 2.6. Statistical Analysis

Calculation of sample size was based on the mean of VAS scores reported in a previous study of 12-week yoga intervention [41]. Our planned sample size of 14 had 80% power to detect a pre-post difference of 10.7 mm on the VAS [41] assuming a two-sided *α* = 0.05, 0.8 standard deviation and 20% dropout rate. An independent *t*-test was used to compare continuous variables at baseline between groups. A two-way ANOVA with repeated measures including time (baseline versus week 12) and group (yoga group versus control group) as factors was used to test whether or not the yoga group experienced significant difference in pain, BDNF, serotonin, flexibility, and depression compared with the control group. When interactions of group by time were significant, paired *t*-tests were performed in each group to determine statistically significant differences between baseline and week 12 in all outcomes. All data were analyzed using SPSS version 13.0 (SPSS Inc., Chicago, IL, USA). The significant level was set at 0.05 for all analyses.

## 3. Results

### 3.1. Baseline Characteristics

General characteristics at baseline in the yoga (*n* = 14) and the control (*n* = 11) groups are presented in Table 1. Duration of back pain was classified into three categories: less than 3 years (*n* = 9); 3–5 years (*n* = 4); and more than 5 years (*n* = 12). One person was a smoker. All subjects were not on any medication except two participants who were taking anemia medications. None of the subjects were presently engaged in regular exercise or other therapies for back pain. Past uses of treatment for low back pain in the previous 12 months were acupuncture (*n* = 8), physical therapy (*n* = 6), and exercise therapy (*n* = 1). No significant differences between two groups were present in age and BMI at baseline.

### 3.2. Primary Outcome

No significant differences in VAS and serum BDNF and serotonin levels between the yoga and the control groups were observed at baseline. Results of VAS and serum BDNF and serotonin levels are presented in Table 2. Analysis with repeated measures 2-way ANOVA revealed that group × time interactions were significant in all outcomes including VAS (*P* < 0.001) and serum BDNF (*P* < 0.01) and serotonin (*P* < 0.05). These results indicate that the changes from baseline at 12 weeks observed in the yoga group were significantly different from those in the control group. Significance of the differences from baseline was tested by paired *t*-test in each group. After 12-week yoga intervention, VAS (*P* < 0.001) significantly decreased in the yoga group, whereas it significantly increased (*P* < 0.05) in the control group. Serum BDNF significantly increased in the yoga group (*P* < 0.01), whereas it tended to decrease in the control group (*P* = 0.052). Serum serotonin did not change significantly in the yoga group while serum serotonin reduced significantly in the control group (*P* < 0.01).

### 3.3. Secondary Outcomes

No significant differences in back flexibility and BDI between the yoga and the control groups were observed at baseline. Table 2 shows that group × time interactions were significant in back flexibility (*P* < 0.05) and BDI (*P* < 0.05). Back flexibility significantly increased in the yoga group during trunk flexion (*P* < 0.01). But there was no significant difference in the control group. BDI did not change significantly in the yoga group and tended to increase in the control group (*P* = 0.072).

## 4. Discussion 

The primary finding of the present study was yoga intervention decreased back pain, accompanied by increasing serum BDNF level in premenopausal women with chronic low back pain. Subjects in the yoga group demonstrated a significant decrease in back pain intensity measured by VAS and a significant increase in flexibility after 12-week yoga intervention whereas back pain increased over 12 weeks in the control group, indicating yoga-induced improvements in back pain and physical function. This finding is consistent with results from previous studies that reported improvements in pain intensity and flexibility in patients with back pain following yoga intervention [35, 41, 42].

In the present study, we found that serum BDNF level significantly increased after 12-week yoga intervention. Although the effect of changes in circulating BDNF on pain has not been studied, beneficial effects of exercise are relatively well known to be mediated by increased circulating BDNF in healthy subjects and patients with other diseases [20, 27–29]. In addition, it has been previously implicated that BDNF plays a role in nociception of back pain [23–26]. Therefore, we hypothesized that beneficial effect of yoga on back pain may be associated with changes of circulating BDNF level. Here, we show that serum BDNF level significantly increased along with decreased pain after yoga intervention in patients with chronic low back pain, suggesting an antinociceptive effect of BDNF on chronic low back pain.

It is not yet fully understood whether BDNF is exclusively nociceptive or antinociceptive [43]. Some studies suggested that BDNF modulates inflammatory pain hypersensitivity in animal studies [21, 44]. However, increased BDNF level with induced pain could be interpreted by a compensatory mechanism to inhibit pain perception. Indeed, it has been suggested that high levels of circulating BDNF presumably have a predominant antinociceptive effect [21, 45, 46]. Regarding plausible connection between the effect of yoga and circulating BDNF level, only one study showed that, in depression patients, the 12-week yoga intervention increased BDNF along with decreased severity of depression [30], which is consistent with the results of the present study.

Our results also demonstrated that serum serotonin level significantly decreased with a significant increase in the back pain intensity in the control group, whereas it maintained in the yoga group. We speculate that, in the control group, the increased pain perception in patients with chronic low back pain may be at least in part due to the decreased serotonin level, and in the yoga group, serum serotonin level did not decrease because yoga intervention may upregulate and/or stabilize the serotonin level over 12 weeks.

It has been established that serotonin increases with exercise intervention, mediating beneficial effects of exercise on mental and neurological disorders [20, 47]. Serotonin-norepinephrine reuptake inhibitors (SNRIs), commonly used in the treatment of depression, have been also considered as pain relievers because those drugs can reduce pain as well as psychiatric symptoms by enhancing serotonin and norepinephrine levels [19, 48, 49]. Interestingly, it has been suggested that serotonin may act cooperatively with BDNF: BDNF enhances serotonin signaling, which in turn increases the level of BDNF [20, 47]. Moreover, it was also reported that serotonergic mechanisms may be involved in the antinociceptive effect of BDNF [50]. Given the proposed functional interaction between BDNF and serotonin in nociception, the involvement of BDNF and serotonin in yoga-induced improvements may at least partly explain the underlying mechanism of beneficial effect of yoga intervention on chronic low back pain.

It is worthy to note that chronic low back pain is suggested to be associated with hormonal and reproductive factors [51]. However, different results have also been reported. Martin [52] reported that hormonal fluctuations during the menstrual cycle may be associated with a mild to moderate effect on pain response. Brynhildsen et al. [53] reported that there was no difference in severity of back pain between the different phases of the menstrual cycle. Thus, the effect of the menstrual cycle on pain seems still inconclusive.

The limitations of this study include the small number of sample size and gender bias. In addition, another possible limitation is type of control group used. The observed treatment effects of yoga could be at least in part due to socialization effect of group classes and/or movement based physical activity. Thus, a sham control group for social interaction and an exercise control group will be needed in future studies to better evaluate the effect of yoga on chronic low back pain.

## 5. Conclusions

Our study is the first clinical study showing that yoga intervention has a significant influence on serum BDNF and serotonin levels in patients with chronic low back pain. We propose that these neuromodulators in nociceptive pathway may mediate beneficial effect of yoga on chronic low back pain.

## Figures and Tables

**Table 1:** Baseline characteristics of subjects.

Variable	Yoga group (*n* = 14)	Control group (*n* = 11)
Age (y)	41.9 ± 8.9	45.0 ± 5.2
Body mass index (kg/m^2^)	21.3 ± 2.0	21.5 ± 2.2
Smoking		
No	13 (92.9)	11 (100)
Yes	1 (7.1)	0 (0)
Medicine		
No	13 (92.9)	10 (90.9)
Yes	1 (7.1)	1 (9.1)
Pain duration		
Less than 3 yrs	4 (28.6)	5 (45.5)
3–5 yrs	3 (21.4)	1 (9.1)
More than 5 yrs	7 (50.0)	5 (45.5)
Previous treatment for back pain		
No	6 (42.9)	6 (54.5)
Yes (multiple response)	8 (57.1)	5 (45.5)
(i) Acupuncture	6	2
(ii) Physical therapy	3	3
(iii) Exercise therapy	1	0

Data are presented as mean ± standard deviation or *n* (%).

**Table 2:** Changes in primary and secondary outcomes.

Variable	Group	Baseline	Week 12	2-way ANOVA with repeated measures		
				*F*_time_ (*P* value)	*F*_group_ (*P* value)	*F*_interaction_ (*P* value)
Primary						
Visual analogue scale (mm)	Yoga	48.7 ± 18.2	16.8 ± 13.2∗∗∗	8.5	26.3	23.1
	Control	48.7 ± 12.4	56.5 ± 9.9∗	(0.008)^##^	(<0.001)^###^	(<0.001)^###^
BDNF (pg/mL)	Yoga	23973.6 ± 6996.6	31395.9 ± 7962.7∗∗	0.1	0.0	14.8
	Control	30949.8 ± 11616.6	24422.4 ± 8206.3	(0.807)	(1.00)	(0.001)^##^
Serotonin (ng/mL)	Yoga	153.8 ± 55.5	143.7 ± 61.7	11.0	0.2	5.0
	Control	186.4 ± 86.9	134.2 ± 57.3∗∗	(0.003)^##^	(0.645)	(0.035)^#^
Secondary						
Back flexibility (cm)	Yoga	4.7 ± 9.5	10.9 ± 8.4∗∗∗	15.3	0.02	7.5
	Control	7.7 ± 5.7	8.8 ± 5.2	(0.001)^##^	(0.892)	(0.012)^#^
Beck Depression Inventory (points)	Yoga	5.5 ± 5.1	4.2 ± 5.4	2.0	2.3	6.5
	Control	5.4 ± 3.3	9.8 ± 6.8	(0.172)	(0.146)	(0.018)^#^

Data are presented as mean ± standard deviation. BDNF: brain-derived neurotrophic factor.

*P < 0.05, **P < 0.01, and ***P < 0.001: paired *t*-test within group.

^#^ *P* < 0.05, ^##^ *P* < 0.01, and ^###^ *P* < 0.001: 2-way ANOVA with repeated measures.
